# Relationship between Orexigenic Peptide Ghrelin Signal, Gender Difference and Disease

**DOI:** 10.3390/ijms22073763

**Published:** 2021-04-05

**Authors:** Chihiro Yamada

**Affiliations:** Tsumura Kampo Research Laboratories, Tsumura & Co., Ibaraki 300-1192, Japan; yamada_chihiro@mail.tsumura.co.jp

**Keywords:** growth hormone secretagogue receptor 1a, Ghrelin, gender difference, food intake

## Abstract

Growth hormone secretagogue receptor 1a (GHS-R1a), which is one of the G protein-coupled receptors (GPCRs), is involved in various physiological actions such as energy consumption, growth hormone secretion promoting action, and cardiovascular protective action. The ligand was searched for as an orphan receptor for a while, but the ligand was found to be acylated ghrelin (ghrelin) discovered by Kangawa and Kojima et al. in 1999. Recently, it has also been reported that dysregulation of GHS-R1a mediates reduced feeding in various diseases. On the other hand, since the physiological effects of ghrelin have been studied exclusively in male mice, few studies have been conducted on gender differences in ghrelin reactivity. In this review, we describe (1) the characteristics of GHS-R1a, (2) the role of ghrelin in hypophagia due to stress or anticancer drugs, and (3) the gender differences in the physiological effects of GHS-R1a and the influence of stress on it.

## 1. Ghrelin and Its Receptor, GHS-R1a

Ghrelin is the only hyperphagia hormone that is peripherally produced. This orexigenic peptide hormone is known to have a strong appetite-promoting effect when administered exogenously in pharmacological doses. Ghrelin is a unique peptide that contains 28 amino acids and an *n*-octanoyl group. Activation of ghrelin requires acylation with Serine 3 catalyzed by ghrelin *O*-acyl-transferase. The active form of acylated ghrelin is deacylated by blood and tissue proteases and metabolized to des-acyl ghrelin, which has no feeding activity. Ghrelin stimulates the release of growth hormone [1]. The agonist or antagonist is considered a promising target for cancer-related cachexia/sarcopenia and obesity treatment. The target of the ghrelin ligand is the 7-transmembrane G protein-coupled receptor (GPCR), growth hormone secretagogue receptor 1a (GHS-R1a), which is composed of 366 amino acid residues. Recent elucidation of the crystal structure of the ghrelin receptor has revealed that the wide gap between the TM6 and TM7 bundles (which are rich in hydrophobic amino acids that contain clusters of phenylalanine residues) is characteristic of the ghrelin receptor-ligand binding pocket [2]. GHS-R1a is activated via the activation of certain G proteins (Gα_q_ and Gα_i/o_) and mobilization of β-arrestin after ghrelin binding [3,4,5]. Its actives classical protein kinase C (PKC) cascade, activating adenosine monophosphate (AMP)-activated protein kinase (AMPK). GHS-R1a is a member of the GPCR family [6] and is homo- or heterodimerized [7] with GHS-R1b [8] as inactive splicing variants [9], dopamine receptors [10], and serotonin 2C receptors, such as melanocortin 3 receptors [11]. GHS-R1a is characterized by its complex signaling regulation, which activates several different signaling pathways when it is stimulated with a ligand. GHS-R1a constantly signals cells with 50% of the highest input activity in a process known as constitutive activity [12,13]. GHS-R1a is present in the vagal afferent nerve, spleen, myocardium, bone, fat, thyroid gland, adrenal glands, and pancreatic and immune cells [14,15]. The expression of GHS-R1a in these tissues suggests pharmacological effects other than the action of appetite and metabolism, and many studies have been conducted to study these effects. In the central nervous system (CNS), GHS-R1a is densely expressed in the hypothalamic nucleus [14,15] and is also localized in the ventral tegmental area (VTA) [16], amygdala [17], and hippocampus [18]. GHS-R1a may therefore play an important role in energy metabolism in both peripheral and central tissues [19].

## 2. Peripheral Ghrelin Signaling

Since the discovery of ghrelin, its relationship with appetite regulation has been examined from a wide range of angles. As a result of these studies, the anorexic mechanisms of various diseases have been elucidated, and it has become clear that ghrelin dysregulation plays an important role in disease progression [20,21,22,23,24,25]. Ghrelin is secreted from the gastric fundus around 1 to 2 h before the start of a meal when the blood concentration increases; around 1 h after a meal, its secretion decreases along with the blood level concentration [26]. Thus, it is considered that an increase in ghrelin is one of the indexes that informs the state of hunger toward the CNS. Ghrelin produced by X/A-like cells in the gastric fundic mucosa acts on ghrelin receptors at the ends of nearby vagus nerves to transmit ghrelin signals to the CNS. The solitary tract nucleus (NTS) forms a network region in the efferent and afferent neural pathways and regulates appetite. Appetite regulators such as CCK, peptide YY, and GLP-1 also play important roles in this pathway by regulating ghrelin signals via blood and neural pathways. Peripheral ghrelin administration increases the levels of c-fos protein, an indicator of neural activity in the NTS [27,28]. The NTS then converts peripheral signals into noradrenergic stimuli and transmits them to the CNS [29]. The ghrelin signal is inputted into the arcuate nucleus of the hypothalamus via the NTS. Ghrelin transmits electrical signals to neuropeptide Y (NPY) and agouti-related peptide (AgRP) neurons, stimulating appetite-promoting peptide neurons and promoting NPY and AgRP synthesis [30,31].

Proopiomelanocortin (POMC), a peptide that is synthesized by appetite-suppressing POMC neurons in the arcuate nucleus, antagonizes AgRP and melanocortin 3,4 receptors in the paraventricular nucleus, and NPY may suppress the appetite-suppressing activity of POMC. In addition, ghrelin signals suppress the secretion of peripheral anorexic hormones, such as CCK and GLP-1 [32].

## 3. Other Central Ghrelin Signaling

Ghrelin administered into the brain also induces a strong appetite-promoting effect. Direct microinjection of ghrelin into the arcuate nucleus induces strong feeding behavior [33], suggesting an important role of ghrelin in the feeding-promoting action in the arcuate nucleus. GHS-R1a is also abundant in the AgRP neurons of the arcuate nucleus [31], and ghrelin may play a role as a neurotransmitter. In addition, ghrelin released into the blood can be found in the unprotected blood-brain barrier and the relatively loose blood-cerebrospinal fluid (CSF) barrier in the choroid plexus. It has also been reported that it may pass directly from the blood and be transferred into the brain. From these findings, it is considered that ghrelin acts on the CNS via a route that differs from that of the signal produced in the peripheral vagus nerve. However, the mechanism by which the ghrelin is selectively transferred from the periphery to the CNS and reaches the central GHS-R1a expression site has not yet been elucidated. Interestingly, GHS-R1a is also expressed in the VTA and reward system circuits such as the nucleus accumbens, the main projection site, and the hippocampus [16,34,35,36]. GHS-R1a regulates the mesolimbic dopaminergic reward circuit [34], and it has been reported that direct administration of ghrelin to the VTA causes an increase in food intake and that administration of an antagonist relieves the hyperphagia caused by intraventricular administration of ghrelin [34,37]. Activation of GHS-R1a localized in the hippocampus induces feeding behavior [38,39]. In addition, in the anterior pituitary gland, although growth hormone-releasing hormone (GHRH) is known as a typical growth hormone-secreting hormone [40], GHSR-1a may also stimulate the release of growth hormone. It is possible that the hypothalamus activates dopaminergic nerves in the mesolimbic system to convey peripheral hunger to central nerves, thereby motivating them to move into eating behavior. In the hippocampus, since ghrelin has been reported to enhance neurogenesis, memory, and cognitive abilities [18,41], it is likely that it is involved in food memories, such as taste and smell.

## 4. Abnormal Ghrelin Secretion and Changes in Reactivity

It is known that, under normal conditions, ghrelin production in the stomach and secretion into the blood are decreased during satiety, while production increases during fasting and starvation, resulting in an increase in ghrelin concentration in the blood [26]. Conversely, blood ghrelin concentration and appetite have been found to be decreased in patients who have been administered with anticancer drugs, patients with gastric mucosal atrophy or gastrectomy [42,43,44,45], and in animal models of these conditions [22,46,47,48,49]. Blood ghrelin levels have been found to be reduced in mice treated with the anticancer drug cisplatin [46,47] and classical SSRIs [49], as well as during psychological stress [22] and cancer cachexia [48]. More interestingly, in cisplatin-treated rats, reduced hypothalamic ghrelin secretion is also observed [50], so ghrelin depletion in both peripheral and CNS may mediate anorexia. Also, in cachexia patients and animal models, reduced food intake impedes sustained nutrient uptake. The result is a status similar to starvation accompanying the increased peripheral ghrelin. In these diseases, exogenous ghrelin’s hyperphagia effect is diminished, resulting in so-called ghrelin resistance. It may involve intracellular internalization [51] by binding to GHS-R1a and the ligand ghrelin. Administration of ghrelin or GHS-R1a agonists to patients with peripheral or central ghrelin levels improved nausea, vomiting, and anorexia [52,53]. It was suggested that ghrelin may also mediate the decrease in feeding-related to various digestive disorders and side effects of drugs.

Ghrelin secretion is negatively regulated by the activation of the serotonin (5-HT) 2B receptor (5-HT_2B_R) [54] located in the smooth muscle of the stomach and the 5-HT 2C receptor (5-HT_2C_R) [55] located in the CNS and heart. Rikkunshito, a Japanese herbal medicine, is a medical drug licensed by the Ministry of Health, Labor, and Welfare of Japan to exert the pharmacological action of a GHS-R1a agonist and to promote ghrelin secretion in the stomach via 5-HT_2B/2C_R antagonism [46]. It has also been proven to enhance the reactivity of endogenous ghrelin [49,56]. It has been reported that rikkunshito improves appetite loss caused by anticancer drug administration [46,50,57,58], various stresses [59,60,61], and cancer cachexia [48,49]. In addition to GHS-R1a agonists, 5-HT_2B/2C_R antagonist or rikkunshito may also be expected as a therapeutic agent for these diseases.

## 5. Ghrelin-Induced Hyperphagia and Gender Difference

**Figure 1:**
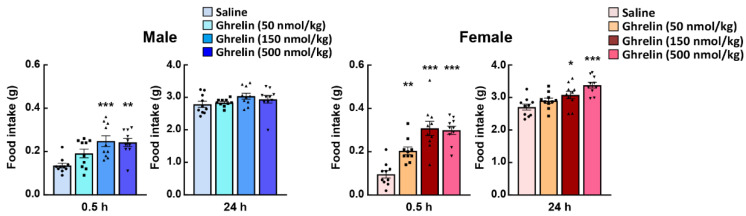
Effect of exogenous acylated ghrelin on the food intake of male and female mice. Ghrelin was intraperitoneally administered to ad libitum-fed mice at the start of the dark period. *, **, *** *p* < 0.05, 0.01, 0.001 vs. saline by Dunnett test. *n* = 10.

In general, the feeding behavior of nocturnal mice shows a clear increase after the start of the dark period, constant feeding behavior is maintained throughout the dark period and then decreases in the light period. Since only male mice have been used to evaluate ghrelin reactivity in most of the studies to date, the effects of gender differences on the physiological effects of ghrelin have not been widely studied. Unfortunately, there are very few studies on the involvement of gender differences in the orexigenic effects of ghrelin administration, except in the basic study by Clegg et al. [62]. Clegg et al. demonstrated the more sensitive appetite-promoting effect on male rats by intraperitoneally administration of ghrelin to rats, using the amount of food consumed one hour later as an index of ghrelin action. Other reports also concluded, based on Clegg’s report, that male rats and untreated ovariectomized females are more responsive to the orexigenic effects of ghrelin than intact or estradiol-treated ovariectomized females [63,64]. On the other hand, intraperitoneal administration of pharmacological doses of acylated ghrelin to 10-week-old C57bl/6J mice at the onset of the dark period was shown to significantly increase food intake within 1 h in female mice [59]. At this time, female mice were found to be more responsive to ghrelin (50–500 nmol/kg, i.p. administration) to feeding behavior than male mice. Moreover, 24 h after ghrelin administration, the feeding levels in male mice returned to almost the same levels as those in the control (saline administration) group; but, in female mice, the increase in food intake continued even after 24 h (Figure 1). In addition to the feeding-enhancing action of ghrelin administration, the growth hormone secretory action, a typical physiological action of ghrelin, is also higher in female mice [59]. However, there were some protocol differences between these studies, such as species difference, with/without surgery, significant weight differences between male and female mice, and the way the effects of ghrelin were measured in the acute phase after administration.

**Figure 2:**
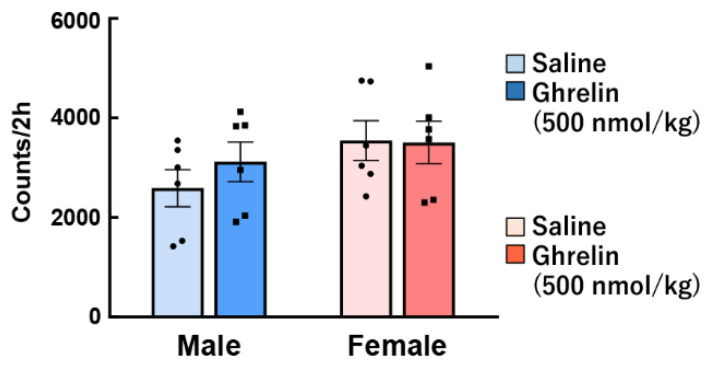
Effect of ghrelin on voluntary movements in male and female mice. Ghrelin (500 nmol/kg) was intraperitoneally administered to ad libitum-fed mice at the start of the dark period, and then the total locomotor activity over 2 h was evaluated. *n* = 6.

**Figure 3:**
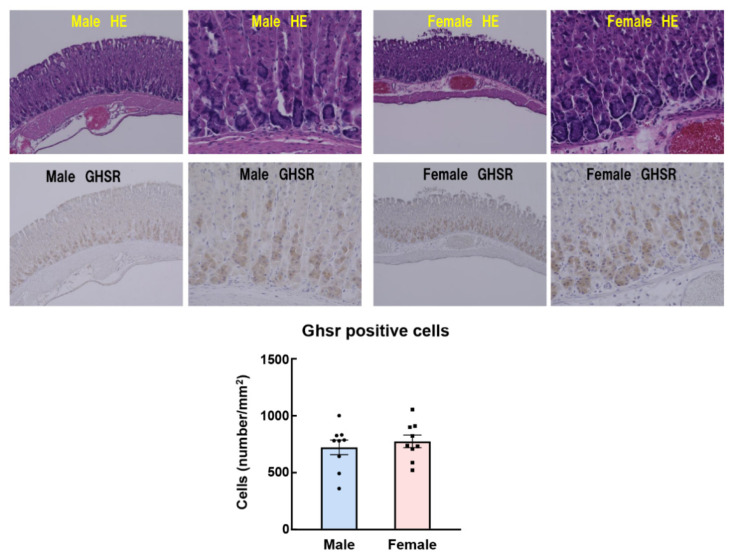
Immunohistochemical staining of Ghsr positive regions in the gastric mucosa and number of Ghsr positive cells among gastric chief cells. The upper row shows hematoxylin eosin (HE) staining, and the lower row shows Ghsr stained (Anti-Ghrelin Receptor antibody ab95250, abcam) photographs. The figure shows the number of positive cells per mucosal region (mm^2^). *n* = 9.

Another study showed that ghrelin injection directly to the hypothalamus has no sex difference with regard to food intake and energy metabolism [65]. Interestingly, we have shown that the orexigenic action by intraperitoneal administration of ghrelin was noticeably higher in female mice than male, but not observed gender difference by intraventricular administration [59]. It is possible that there is a gender difference in ghrelin signal transmission from the periphery, although there is no gender difference in ghrelin reactivity in the brain. However, the detailed reason for this discrepancy is unknown, and further research and discussion will be needed in the future. The data from these previous studies show the responses to exogenous intraperitoneally administered ghrelin by pharmacological load, and the action on endogenous ghrelin was not investigated. Some studies have reported that plasma ghrelin levels are higher in women than in men [66,67]. It also demonstrated that peripheral ghrelin after a 12-h fast was clearly higher in female rats than in males [68]. In contrast, others have reported no differences in plasma ghrelin levels between males and females [69]. Differences in endogenous ghrelin concentration [59], locomotor activity (Figure 2), number of cells expressing GHS-R1 protein in the stomach (Figure 3), gene expression of preproghrelin (data not shown) in the stomach, and blood dynamics of exogenous ghrelin were not observed in our study [59], suggesting that gender differences in ghrelin reactivity may be involved in receptor affinity and ghrelin signal transmission for appetite.

**Figure 4:**
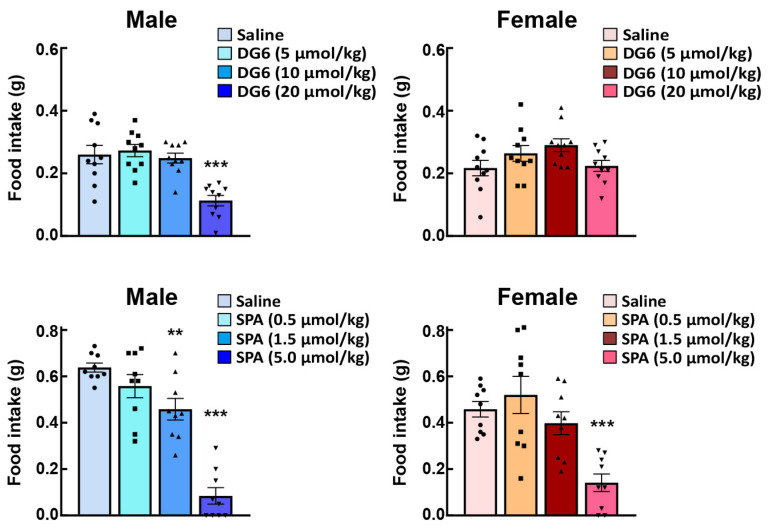
Effects of intraperitoneal administration of GHSR antagonists on the food intake of male and female naïve mice. The upper row shows the effects of (D-Lys^3^)-GHRP-6 (DG6) on 2-h food intake, and the lower row shows the effects of (D-Arg^1^, D-Phe^5^, D-Trp^7,9^, Leu^11^)-substance P (SPA) on 2-h food intake. **, *** *p* < 0.01, 0.001 vs. saline by Dunnett test. See the ghrelin administration section for a dosing schedule. *n* = 9–10.

After ghrelin administration, neural activity (indexed by c-fos expression in the NTS, arcuate nucleus, and paraventricular nucleus) and AgRP gene expression in the arcuate nucleus were enhanced in female mice as compared with their male counterparts [59]. These results may suggest that exogenously administered ghrelin signals are enhanced prior to reaching the NTS in female mice. In order to investigate the reactivity of endogenous ghrelin, a study involving the administration of a ghrelin receptor antagonist to normal male and female mice is required. GHS-R1a has constitutive activity [70] and signals 50% of the maximum input [12,70,71]. Previous studies have shown that peripheral and intracerebral administration of the ghrelin antagonist, (D-Lys^3^)-GHRP-6, suppresses food intake with or without ghrelin administration [72,73]. Intraperitoneal administration of (D-Lys^3^)-GHRP-6 [13,74], which cannot suppress all of this constitutive activity, was observed to reduce food intake in male mice (Figure 4 upper). Conversely, no effect was observed in female mice. When the inverse agonist (D-Arg^1^, D-Phe^5^, D-Trp^7, 9^, Leu^11^)-Substance P (SP-analog) [71,75] were administered to male or female mice, a decrease in food intake was confirmed from lower doses in male mice (Figure 4 lower row); however, exposure to the same amount of antagonist failed to suppress the endogenous ghrelin signal in female mice. Figure 3 shows the immunostaining of Ghsr in the gastric fundus. Although there are still some issues regarding the specificity of the antibody against Ghsr in the immunostaining method, there was no difference in the Ghsr protein expression positive area (%) in the gastric fundus between males and females. In addition, GHS-R1a mRNA expression in the fundus is extremely low; therefore, the fundus may not be the GHS-R1a synthesis site. It is likely that female mice have a higher affinity for ghrelin and GHS-R1a than male mice, or that endogenous ghrelin may have higher post-binding GHS-R1a signaling in females.

In rats and mice, the gonadal steroid hormone, estrogen, is known to regulate feeding. Estrogen depletion has been shown to clearly increase feeding behavior [59,76,77]. Clegg et al. [62] found that food intake by intraperitoneal and intraventricular (third ventricle) ghrelin was significantly higher in ovariectomized rats than in sham-operated female rats or ovariectomized rats treated with estradiol. Estrogen α receptor (ERα) is expressed in rat gastric mucosal cells [78] and may regulate ghrelin production in the stomach. Ovariectomy temporarily increases both ghrelin-positive cell numbers and plasma ghrelin levels [78]. Therefore, in female mice, the ghrelin signal is always negatively regulated by estrogen.

In the 1990s, two types of nuclear ER (ERα and ERβ) were identified [79,80]. ERα knock-out male and female mice tended to be obese, and increased energy consumption, body weight, and lipids were also observed [81,82,83]. In addition, ERα agonist administration suppressed feeding in ovariectomy and normal mice [84,85]. ERα plays a central role in the negative control of estrogen-related feeding behavior. This suppression of feeding is mainly confirmed in the CNS [64], not in the peripheral area. Of the NTS that relay the ghrelin signal from the periphery to the CNS, the caudomedial nucleus of the NTS (cmNTS) has the densest population of ERα-expressing neurons [86,87]. Estradiol enhances the feeling of fullness caused by CCK. After CCK administration, estrogen administration supports the enhancement of NTS neural activity in rodents [88,89]. In this way, the anorexic effect of ERα in the NTS seems to be closely related to the effect of CCK. Further studies are needed to clarify whether ghrelin directly or indirectly suppresses the ERα-operated nervous system. Studies into the differences in ghrelin responsiveness between mice and humans and between the different genders will reveal more detailed mechanisms.

## 6. Stress Load and Gender Differences

Abnormal appetite during stress is a symptom that is easily observed around us. In previous studies, stress has been shown to cause ghrelin dysregulation, and relatively mild and chronic stress is known to stimulate eating and lead to binge eating [90,91,92]. On the other hand, stress is also a cause of anorexia [21,22,23,24]. Decreased peripheral ghrelin levels may directly suppress the transmission of peripheral hunger signals. On the other hand, relatively mild stress exposure results in so-called ghrelin resistance, when an increase in feeding behavior does not occur despite an increase in peripheral ghrelin [56,59,93]. In normal female mice, the electrical stimulation of nerves from the NTS to the arcuate nucleus has been shown to be clearly higher than that in males after ghrelin exposure, and AgRP gene expression in the hypothalamus has been shown to be enhanced [59]. This effect is due to the fact that untreated naïve female mice also have high NPY/AgRP gene expression in the hypothalamus. Female mice may be more susceptible to ghrelin signals or less susceptible to inhibitory signals than male mice. Interestingly, the high ghrelin responsiveness in female mice has recently been demonstrated to be canceled under stress loading [59]. In addition, stress has been shown to reduce post-ghrelin-loaded neural activity in the NTS of the medulla oblongata, the relay point for ghrelin signals. The ERα is expressed in the NTS and arcuate nucleus [64]. The expression of this receptor is enhanced by stress, while the neural activity of neurons that have this receptor is also enhanced. Furthermore, administration of α-receptor antagonist instead of ERβ to stressed mice has been found to restore decreased feeding to almost normal levels. These features may play major roles in the mechanisms of reduced food intake under stress loading in female mice. Studies into acute psychological stress in rodents may provide hints for women who have high levels of depression and neuropathic anorexia caused by stress-based background factors.

## 7. Conclusions

Maintaining feeding in refractory diseases is crucial in nutrition, but the mechanism remains unclear. Appetite is under complex regulation by many appetite-related peptides. One of its key factors is ghrelin. Recent findings have found that loss of appetite is mediated by abnormal production and transmission of appetite-related peptides such as ghrelin. More important findings indicate that there may be gender differences in ghrelin reactivity. Gender-specific differences in ghrelin responsiveness may also be related to the fact that ghrelin may mediate part of a woman’s fertility [94,95]. The ghrelin-induced appetite-promoting effect of female mice may be important for maintaining fertility. Further research in this area is needed. In addition, antitumor loading is one of the most intense stresses, and malnutrition due to loss of appetite reduces the patient’s quality of life. The vulnerability to eating disorders in relation to stress in women can be mediated by ghrelin transmission disorders. The administration of ghrelin and drugs that promote ghrelin transmission may be an effective means of treating eating disorders in a variety of stress-induced disorders in women.

## Data Availability

Data available on request. The data presented in this study are available on request from the corresponding author.
